# What challenges still exist in the critical care of children?

**DOI:** 10.1186/s12887-022-03649-9

**Published:** 2022-10-13

**Authors:** Fola Odetola, John Pappachan

**Affiliations:** 1 Department of Pediatrics, University of Michigan, Ann Arbor, USA; 2 Southampton Childrens Hospital, Southampton, UK

Paediatric Intensive Care provides organ-specific support to critically ill children with a very broad range of diagnoses. A relatively new specialty, its birth followed the emergence of adult intensive care and was necessitated by developments in neonatal intensive care (that produced long term survivors with ongoing needs for critical care), paediatric cardiac surgery, and anaesthesia. The first paediatric intensive care unit (PICU) was established in Sweden in Europe in 1955, ten years before the unit at Children's Hospital of the District of Columbia was developed [1, 2]. There are now an estimated over 300 PICUs in the United States alone [3].

Multiple quality of care factors in PICU including the existence of dedicated training programs have had a significant impact on the survival of children with critical illness [4]. Child mortality from critical illness or injury is lower in settings where a PICU is available [5] and has decreased from 1 in 5 children in the early 1990s [6], to less than 5% currently, according to recent estimates [7].

The exponential growth in pediatric intensive care research (1 citation under PICU in 1979 in PubMed compared with 904 in 2020), the development of training programmes, and advancements in technology, diagnostics and surgical/anaesthetic techniques have contributed to a remarkable reduction in unadjusted and case-mix adjusted PICU mortality, but challenges and unmet needs still exist in the critical care of children as highlighted below.

## SEPSIS

The development of the specialty has allowed for paediatric-specific definitions and a multi-disciplinary approach to the care of children with sepsis using care bundles that incorporate evidence-based approaches to ventilation, fluid resuscitation, inotropic support, and extracorporeal membrane oxygenation (ECMO) for refractory cases. This evidence-based care has been associated with improved mortality [8]. Recent international data suggest however, that although the prevalence of severe sepsis has fallen to 8.2%, the mortality in both industrialised and non-industrialised nations remains at 25% [9]. The COVID-19 pandemic has created a legacy of new research re-exploring the role of targeted immunomodulation in adults and in children with the Multi System Inflammatory Syndrome (MIS-C) [10] The future may include research exploring the use of immunomodulatory strategies in cytokine storm syndromes including severe sepsis in children.

## Paediatric Acute Respiratory Distress Syndrome (PARDS)

Improvements in outcomes in adults with acute respiratory distress syndrome (ARDS) with the adoption of low tidal volume ventilation [11] and prone ventilation [12], has not been replicated in children, with mortality in severe PARDS remaining high at 33% [13]. Further, 27% of children with PARDS are managed with a level of PEEP lower than that recommended by adult guidelines, a gap in practice that is independently associated with mortality [14]. Importantly, non-adherence to lung-protective ventilation principles in PARDS has been associated with adverse outcomes [15]. These findings suggest an urgent need to close the gap between evidence and practice to enhance the outcomes for children with PARDS.

## Traumatic Brain Injury (TBI)

TBI is one of the leading causes of death and long-term disability across all ages worldwide, but children and the elderly are the most at risk. Unlike the elderly, the burden of disability in children is life long and thus has far greater socioeconomic impact. The care delivered in PICU to children with TBI is limited by the poorly described but important anatomical and physiological differences between the adult and immature brain and despite this, the need to extrapolate adult data to the paediatric population because of the lack of paediatric-specific TBI research. A pragmatic update to the Brain Trauma Foundation Guidelines was published in 2019 and contained 22 recommendations, however, none were based on Level I data and only three incorporated Level II data [16]. Approaches and Decisions for Acute Pediatric TBI Trial (ADAPT) [17], a multi-national comparative effectiveness research study is under way but is likely to generate more questions than answers.

## Neurodevelopmental comorbidity

Critically ill children requiring PICU admission increasingly survive but with multimorbidity. Post-intensive care syndrome-pediatrics (PICS-p) includes abnormalities in neurocognition, physical and social scores, and health-related quality of life. Two recent reports [18, 19] have been published which raise awareness of this unresolved issue and establish a core set of outcome measures to enhance quantification of the effects of PICS-p across multiple domains. 50% of bed days in the UK are now occupied by only 10% of admissions comprised of children who have neurodevelopmental comorbidity and are often technology dependent. Moreover, many children hospitalized with critical illness have baseline developmental impairment which heightens the risk for critical illness, death and prolonged PICU stay, making them among the most vulnerable PICU patients.

## Management of sedation, withdrawal and delirium

We still have a long way to go in terms of understanding the optimal management of sedation, iatrogenic medication withdrawal syndrome, and delirium in children who survive critical illness. These issues have been extensively studied in adults but less so in children in whom an additional burden of the longer-term effects of sedation on a developing brain may be much more important [20].

The survivors of critical illness often have long term neurodevelopmental morbidity and technology dependence. In addition, the availability of novel therapies for previously fatal diseases and the availability in many countries of long term ventilatory support has meant that many more technology dependent children are surviving and being admitted to PICU. There is, therefore, an urgent need to enhance our understanding of disease pathogenesis and of innovative approaches to improve diagnosis and treatment. This call for articles also seeks to discover new approaches to multidisciplinary clinical care of children in the hospital setting while appreciating the need for disease prevention and the value of post-hospital care of critically ill children. We would welcome articles dealing with basic and translational science, clinical care and health services and systems research. 

The Journal is keenly interested in research articles that report findings from randomized controlled trials, trial protocols, and observational studies. Review articles on important issues in the care of critically ill children requiring PICU will also be considered.
